# The anticoagulant properties of cadmium telluride quantum dots

**DOI:** 10.1002/jin2.35

**Published:** 2018-04-16

**Authors:** Ciarán M. Maguire, Michelle Lavin, Mairead Doyle, Mary Byrne, Adriele Prina‐Mello, James S. O'Donnell, Yuri Volkov

**Affiliations:** ^1^ School of Medicine, Trinity Translational Medicine Institute, Trinity College Dublin Dublin Ireland; ^2^ AMBER Centre, CRANN Institute, Trinity College Dublin Dublin Ireland; ^3^ National Coagulation Centre, St. James's Hospital Dublin Ireland; ^4^ Irish Centre for Vascular Biology, Royal College of Surgeons in Ireland Dublin Ireland; ^5^ International Laboratory of Magnetically Controlled Nanosystems for Theranostics of Oncological and Cardiovascular Diseases ITMO University St. Petersburg Russia

**Keywords:** Blood coagulation factors, platelet function tests, Von Willebrand factor, quantum dots

## Abstract

The size‐dependent optical properties of quantum dots (QDs) are frequently exploited for use in medical imaging and labelling applications. Similarly, presented here, they also elicit profound size‐dependent anticoagulant properties. Cadmium telluride quantum dot (QDs) (3.2 nm) were shown to have a dramatic anticoagulant effect centred on around the intrinsic coagulation pathway, compared to their 3.6 nm counterparts. Several clinically relevant diagnostic tests were carried out over a concentration range of the QDs and demonstrated that the 3.2 nm QDs elicited their response on the intrinsic pathway as a whole, yet the activity of the individual intrinsic coagulation factors was not affected. The mechanism appears also to be strongly influenced by the concentration of calcium ions and not cadmium ions leached from the QDs. Static and shear‐based primary haemostasis assays were also carried out, demonstrating a profound anticoagulant effect which was independent of platelets and phospholipids. The data presented here suggest that the physical–chemical properties of the QDs may have a role in the modulation of haemostasis and the coagulation cascade, in a yet not fully understood mechanism. This study has implications for the use of similar QDs as diagnostic or therapeutic tools in vivo, and for the occupational health and safety of those working with such materials.

## Introduction

Semiconductor nanocrystals or quantum dots (QDs) are the focus of extensive research in the field of nanoscience due to their unique physical properties and have a wide range of potential applications in photonics, solar cells, biotechnology, medicine and imaging (Maguire et al., 2014). The biomedical applications of cadmium telluride (CdTe) QDs have been demonstrated by various groups (Gerard et al., 2011; Maguire et al., 2014; Prasad et al., 2010; Smith et al., 2008; Wang et al., 2009; Zeng et al., 2009). Typically, the use of QDs in biological studies is focused around the area of tumour cell labelling, particularly due to their highly luminescent properties. Modifications to the QDs can also allow for labelling and differentiation of numerous cell types, similar to the use of fluorescent dyes in flow cytometry, for example (Ibanez‐Peral et al., 2008).

Utilisation of QDs in the clinical setting is hampered by the potential cytotoxicity of QDs, which is thought to arise from the toxicity of the cadmium atoms, photochemical generation of free radicals and nanoparticle aggregation on the cell surface (Ipe et al., 2005; Kirchner et al., 2004). Whilst cytotoxicity may prove beneficial in the selective killing of cancer cells (Lovric et al., 2005), the refinement of targeting of QDs to malignant cells is ongoing to minimise the risk of side effects including apoptosis of healthy cells. Production of QDs has also increased in the last number of years due to their utilisation in electronics and consumer devices such as televisions. This also leads to concerns for those involved in their manufacture, and end‐of‐life disposal of QD containing devises.

Regardless of the route of delivery, whether through intentional clinical intravenous injection for diagnostic imaging or cancer treatment, or accidental pulmonary exposure during manufacturing or disposal, for example, QDs at some point may enter the blood stream, where they will encounter its various constituents, including components of the coagulation cascade, the pathway responsible for maintaining the pro‐thrombotic and anti‐thrombotic balance, crucial for haemostasis. These mechanisms of exposure may inevitably lead to the commonly referred to toxicological responses such as chronic immunotoxicity or genotoxicity. However, to date, few studies to date have investigated the acute impact of QDs on this pathway (Geys et al., 2008). Mice injected with amine and carboxyl modified cadmium selenide/zinc sulphide (CdSe/ZnS) QDs, approximately 2.4 nm in diameter, have been reported to experience pulmonary vascular thrombosis and almost immediate death (Geys et al., 2008). It was also demonstrated that carboxyl modified QDs were more potent than their amine counterparts at inducing pulmonary vascular thrombosis. The authors suggest that the carboxyl QDs activate the coagulation cascade through the contact activation pathway, as the thrombotic effect was not detected when mice were pre‐treated with heparin (Geys et al., 2008), a well‐characterised anticoagulant.

**Figure 1:**
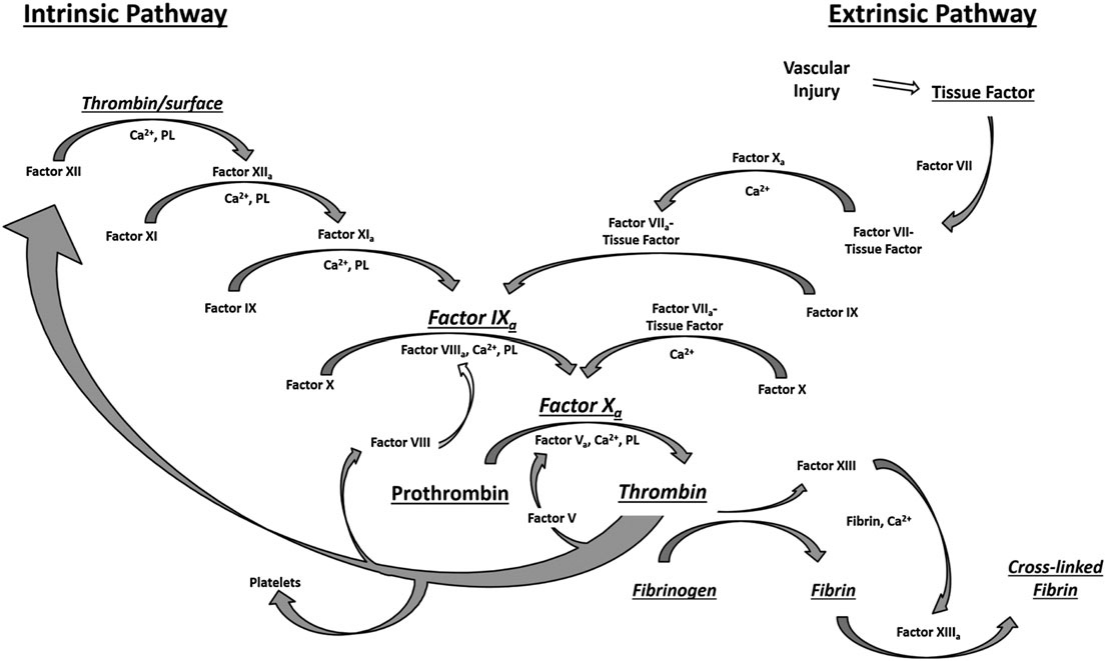
The coagulation cascade is the pathway is responsible for maintaining the balance between a pro‐thrombotic and anti‐thrombotic state. Contact with a negatively charged surface or following vascular injury results in the release of tissue factor, platelet activation and activation of Factor X, followed by the conversion of fibrinogen to fibrin and the formation of a cross‐linked fibrin clot. Feedback loops cause amplification of factor activation. Ca^**2**+^: calcium ions; PL: phospholipids.

The coagulation cascade is a complex pathway resulting in thrombus formation and cessation of blood loss following vascular injury (Kirchner et al., 2004). Typically, blood vessel injury results in exposure of sub‐endothelial collagen and tissue factor, initiating activation of clotting factors, platelets and generation of thrombin with the conversion of soluble fibrinogen to an insoluble fibrin clot (Geys et al., 2008; Lovric et al., 2005), as depicted in Figure 1. Traditionally, there are two main pathways in the coagulation cascade, the intrinsic pathway and extrinsic pathway, which converge to form the common pathway (Kanaji et al., 2012). Collagen binding and platelet function are also crucial for the cessation of bleeding. Under shear stress in the blood vessels, the circulating glycoprotein von Willebrand factor (vWF) becomes stretched exposing a collagen binding site in the A1 domain for the GP1b receptor of the activated platelets (Kanaji et al., 2012). Similarly, platelet adhesion glycoprotein GPIIb‐IIIa mediates the binding of platelets to fibrinogen and VWF and aids in platelet activation and is critical in thrombus formation. Although GPIIb‐IIIa is present on the surface of unstimulated platelets, it only binds fibrinogen after activation (Isenberg et al., 1987). This entire process facilitates the formation of the platelet plug at the site of the injury by forming a network of crosslinked fibrin fibres and aggregated platelets.

Although these pathways are not so clearly distinguishable in vivo, they are still commonly referred to as they form the basis of important tests for coagulation disorders (Cochrane et al., 1973; Isenberg et al., 1987). In the extrinsic pathway, tissue factor released due to vascular damage, binds to Factor VII, which initiates a cascade whereby Factor X is activated, leading to the production of thrombin from prothrombin. The activity of this pathway is measured through the prothrombin time (PT) test. The intrinsic pathway (contact activation pathway) is measured using the activated partial thromboplastin time (APTT) test and requires a contact activator such as kaolin to initiate the APTT (Cochrane et al., 1973). This pathway is sensitive to changes in levels of fibrinogen, thrombin and coagulation factors V, VIII, IX, X, XI and XII. The thrombin generated through the extrinsic and intrinsic pathways then goes on to cleave soluble fibrinogen to insoluble fibrin, which ultimately becomes crosslinked into a stable fibrin network by Factor XIII_a_.

Herein, we report on the impact of CdTe QDs on the coagulation cascade through extensive coagulation testing and present contrasting results to the previous reports. We demonstrate that CdTe QDs can exhibit size‐dependent anticoagulant properties, with 3.2 nm QDs altering coagulation through a yet unknown mechanism. What is evident, however, is that the anticoagulant phenomenon is based on the intrinsic coagulation pathway and that calcium ions concentration plays a crucial role.

## Experimental

### Quantum dot properties

Thioglycolic acid stabilised cadmium telluride (CdTe) QDs were synthesised *via* the aqueous synthesis route, and characterised, by the Prof. Gun'ko Research Group (School of Chemistry, Trinity College Dublin, Ireland) using published procedures (Gaponik et al., 2002; Maguire et al., 2014). Aluminium telluride was purchased from Cerac Inc. (Wisconsin, USA), and all other chemicals for synthesis were purchased from Sigma‐Aldrich (Dublin, Ireland).

In this study, 3.2 and 3.6 nm QDs were utilised, and their physiochemical properties are detailed below in Table 1. A Shimadzu UV‐1601 UV‐Visible Spectrophotometer was used to measure QD absorption, with scans carried out in the 300–700 nm range. A Varian‐Cary Eclipse Fluorescence Spectrophotometer was used to determine the fluorescence emission/photoluminescence spectra of QDs. The excitation wavelength was 480 nm, and the emission was detected in the range 490–700 nm, with the quantum yields being calculated from the photoluminescence spectra using Rhodamine 6G as a reference. Zeta potential measurements were carried out using a Malvern Zetasizer Nano Series V5.10. Five measurements were usually taken for each sample, each made of 10 to 20 accumulations as optimised by the machine. QD sizes were determined using their UV absorbance spectra using previously published methodology (Yu et al., 2003).

**Table 1:** Physiochemical properties of 3.2 and 3.6 nm cadmium telluride quantum dots.

Property	3.2 nm QD	3.6 nm QD
Coating	Thioglycolic acid	Thioglycolic acid
Size (nm)	3.24	3.61
Peak excitation (nm)	550	589
Peak emission (nm)	577	627
Quantum yield	18%	22%
Stock concentration (μM)	50.7	71.2
Zeta potential (mV)	−30	−31

### Ethical approval

Ethical approval for the collection of blood samples from healthy volunteers for use in platelet function, phospholipid and vWF assays was obtained from the local research ethics committee.

### Patient samples

Peripheral blood samples were collected using a 21G needle into 0.109 M trisodium citrate (9:1 *v/v*, BD) from healthy volunteers with no exposure to drugs known to affect platelet function for at least 14 days prior to the study, as per standard practise. Platelet rich plasma (PRP) and platelet poor (PPP) was generated, with PRP used within 4 h of collection and PPP frozen at −80°C prior to use in assays.

### Platelet function

Whole blood samples collected into 0.109 M trisodium citrate were analysed using the PFA‐100 analyser (Dade‐Behring, Marburg, Germany) and the closing times recorded, with and without the presence of the QDs. Concentrations tested were based on previous unpublished results. All findings were compared to control normal reference plasma (Coag‐Norm, Diagnostica Stago, France).

Platelet light transmission aggregometry was carried out on PRP using an Aggregation Remote Analyser Module (AggRAM, Helena Biosciences, UK) using a standard light transmission aggregometry technique. QDs at concentration of 2 and 10 μM were pre‐incubated with platelets for 3 min. Collage (2 μg/mL) was also included as a positive control to induce platelet aggregation, demonstrating that aggregated platelets allow for increased light transmission; 10 μM of QDs was used in this experiment, compared to a maximum concentration of 7.5 μM in the platelet function assay, along with an equivalent volume of water as a control, to maximise the likelihood of observing an effect on platelet aggregation, without excess QDs impeding the light transmission measurement.

The concentration ranges used in the remainder of the study were based on the results obtained from the PFA‐100 assay, where an anticoagulant response was observed with 7.5 μM of the 3.2 nm QDs.

### Von Willebrand factor (VWF) assays

All VWF assays were performed on PPP samples and compared to a reference of pooled normal plasma. VWF Antigen (VWF:Ag) levels were measured using a latex particle enhanced immunoturbidimetric assay (HemosIL VWF antigen assay) on an automated coagulometer (ACL Top 3G, Instrumentation Laboratories, Italy). VWF Ristocetin Co‐factor activity (VWF:RCo) was determined by standard platelet agglutination on a Sysmex CS2100i Analyser. Plasma VWF collagen binding (VWF:CB) was performed on PPP using standard ELISA techniques with a Techonozym collagen III binding ELISA assay kit (Technoclone, Austria). All assays were repeated following incubation of PPP samples with 3.2 and 3.6 nm QDs concentration of 7.5 μM for 1 h at 37°C.

### Coagulation assays

The PT, APTT and thrombin time (TT) assays were performed on KC4 Delta Coagulometer (Amelung, Trinity Biotech, Ireland) on reconstituted normal reference plasma (Diagnostica Stago, France). HemosIL recombiplastin 2G (Instrumentation Laboratory, USA) was used in the PT assay, CK PREST 5 APTT reagent (Diagnostica Stago, France) used in APTT assays, and human thrombin (Sigma Aldrich, Ireland) for the TT. The TT involves the addition of bovine or human thrombin to PPP. It reflects the generation of fibrin but is also sensitive to the presence of inhibitors that may be present in the plasma. Standard techniques were used for the PT (Quick Method), APTT and TT as previously described (Horsti, 2000; Ignjatovic, 2013; Mischke, 2000). DI water at an equivalent volume to the greatest volume of QD added (7.39 μL per 50 μL plasma) was also included as a control.

Intrinsic factor activity (factors VIII, IX, XI, XII) was determined using a one‐stage APTT‐based clotting assay, and extrinsic factor levels (factor II, V,VII, X) were determined using a one‐stage PT‐based clotting assay. For each individual factor assay, PPP samples were mixed with plasma deficient for that factor, and the time for clot formation was recorded using ACL TOP (Instrumentation Laboratories, Italy). For APTT‐based assays, HemosIL SynthASil APTT Reagent and HemosIL SynthASil Calcium Chloride (Instrumentation Laboratories, Italy) provided a source of calcium and phospholipid to activate clotting, and samples were incubated at 37°C for 3 min. All results were compared to HemosIL reference plasma (Instrumentation Laboratories, Italy). To assess the impact of QD on coagulant factor levels, normal reference plasma was incubated with 2 and 7.5 μM QDs for 3 min, followed by a 1/10 dilution in phosphate buffered saline (PBS, Sigma Aldrich, Ireland) to increase the sensitivity of the assay. Subsequently, the QD‐spiked plasma was mixed in 1:1 dilution in factor deficient plasma and each factor assay repeated as above.

### Phospholipid activity

Phospholipid activity was assessed to identify if QDs interact with the function of the phospholipids and assembly of the coagulation complexes using two assays, the dilute Russell's viper venom time (DRVVT) and the silica clotting time (SCT). Briefly, PPP is mixed with two differing concentrations of phospholipid using either the DRVVT reagent (HemosIL, Instrumentation Laboratories, Italy) or SCT reagent (HemosIL, Instrumentation Laboratories, Italy) at 37°C. The APTT was then measured on an ACL Top 3G (Instrumentation Laboratories, Italy) and compared to reference plasma.

### Calcium assay

To identify if cadmium ions leaching from the QDs were interacting with calcium ions required for coagulation to take place, an APTT with varying calcium concentrations (0.025 M CaCl_2_: standard volume: 50 μL; increased volume 100 μL) was conducted on a KC4 Delta Coagulometer (Amelung, Trinity Biotech, Ireland) using reconstituted normal reference plasma (Diagnostica Stago, France) and CK PREST 5 APTT reagent (Diagnostica Stago, France). Volume ratios of plasma to APTT reagent to calcium chloride of 1:1:1 and 1:1:2 were used. Cadmium sulphate (Sigma Aldrich, Ireland) and cadmium chloride (Sigma Aldrich, Ireland) at 7.5 μM, were used as Cd ^2+^ controls to identify if the effect was solely due to cadmium ions.

### Statistical analysis

Data was analysed using GraphPad Prism version 7.00 for Windows (GraphPad Software, La Jolla California, USA) using one‐way and two‐way ANOVAs, with additional multiple comparison tests conducted where appropriate.

## Results and discussion

### Effect of quantum dots on primary haemostasis

#### Platelet function

**Figure 2:**
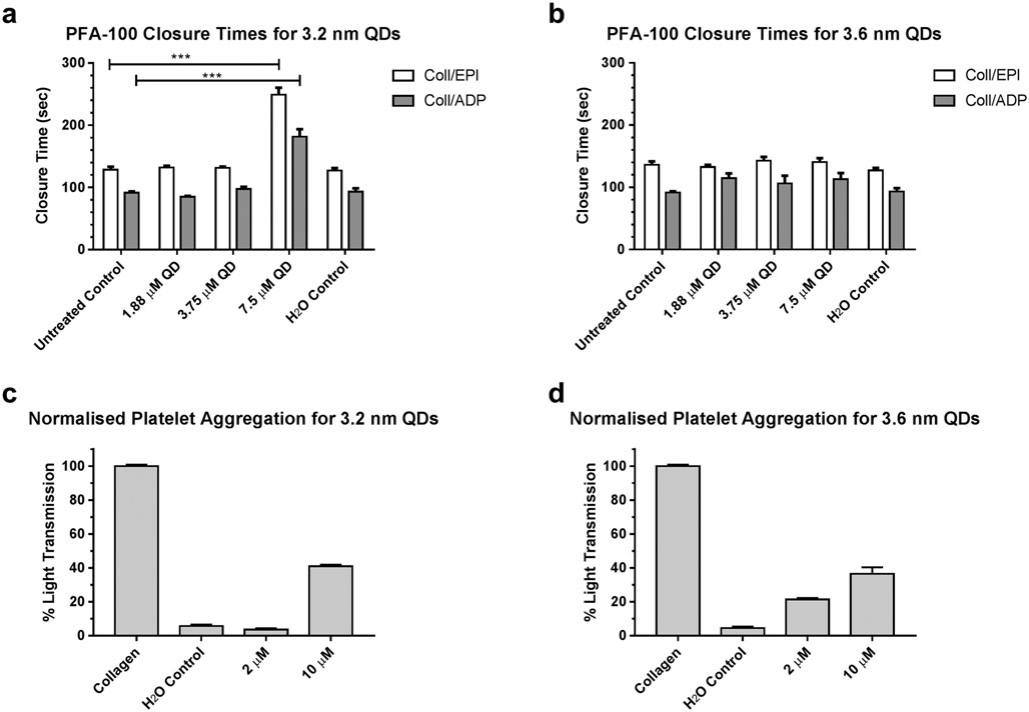
Platelet Function Analyser‐100 (A and B) and platelet aggregation (C and D) results illustrating the size‐dependent effects of quantum dots on primary haemostasis. Aperture closure time is significantly extended outside the normal clinical range at a 7.5‐μM concentration of the 3.2‐nm QDs (A) for both collagen/epinephrine and collagen/ADP (*** *P* = 0.001, one‐way ANOVA with Dunnett's, *n* = 3). No prolongation in closure time is observed at equal concentrations of the 3.6 nm variant (B). Minimal platelet aggregation is observed for both sizes of QDs up to a concentration of 10 μM.

The effect of CdTe QDs on whole blood and platelets was assessed using the PFA‐100 assay and platelet aggregometry. PFA‐100 demonstrates the ability of whole blood to form a platelet plug in a collagen coated aperture in response to activation by epinephrine (Coll/Epi) or adenosine diphosphate (ADP) (Coll/ADP). Using increasing concentrations of 3.2 and 3.6 nm QDs (Figure 2A and B), a significant prolongation in closure times was found in the 3.2 nm QDs at 7.5 μM concentration in comparison to controls (ANOVA with Dunnett's, Coll/ADP: *P* = 0.0001, Coll/Epi: *P* = 0.001). In contrast, no corresponding effect was observed for the 3.6 nm QDs at the same concentration (ANOVA with Dunnett's, Coll/ADP: *P* = 0.1384, Coll/Epi: *P* = 0.8157) suggesting a possible size dependency to the interaction.

Further assessment of platelet aggregation was performed using light transmission aggregometry (Figure 2C and D). Results were normalised to collagen induced platelet aggregation, as collagen is noted as the most thrombogenic component of the sub‐endothelium (Baumgartner & Haudenschild, 1972). Addition of QDs to PRP resulted in minimal platelet aggregation up to a concentration of 10 μM, with a maximum percentage light transmission of 38.6% and 37.1%, for the 3.2 and 3.6 nm QDs, respectively.

From these results, we identified that blood exposure to CdTe QDs results in abnormalities of assays of primary haemostasis. Whilst only the 3.2 nm QDs result in prolongation of the PFA‐100, both 3.2 and 3.6 nm QDs induced minimal platelet aggregation, compared to collagen. These results corroborate with other published results for similar sized QDs, where 2.6 and 4.8 nm CdTe QDs induced minimal platelet aggregation in PRP (Samuel et al., 2015). However, these results differ from other platelet aggregation studies which used QDs synthesised from other materials (Dunpall et al., 2012; Geys et al., 2008; Ramot et al., 2010). These results suggest that nanoparticle material, as well as size, may play a role in the interaction of QDs and haemostasis.

#### Von Willebrand activity

**Figure 3:**
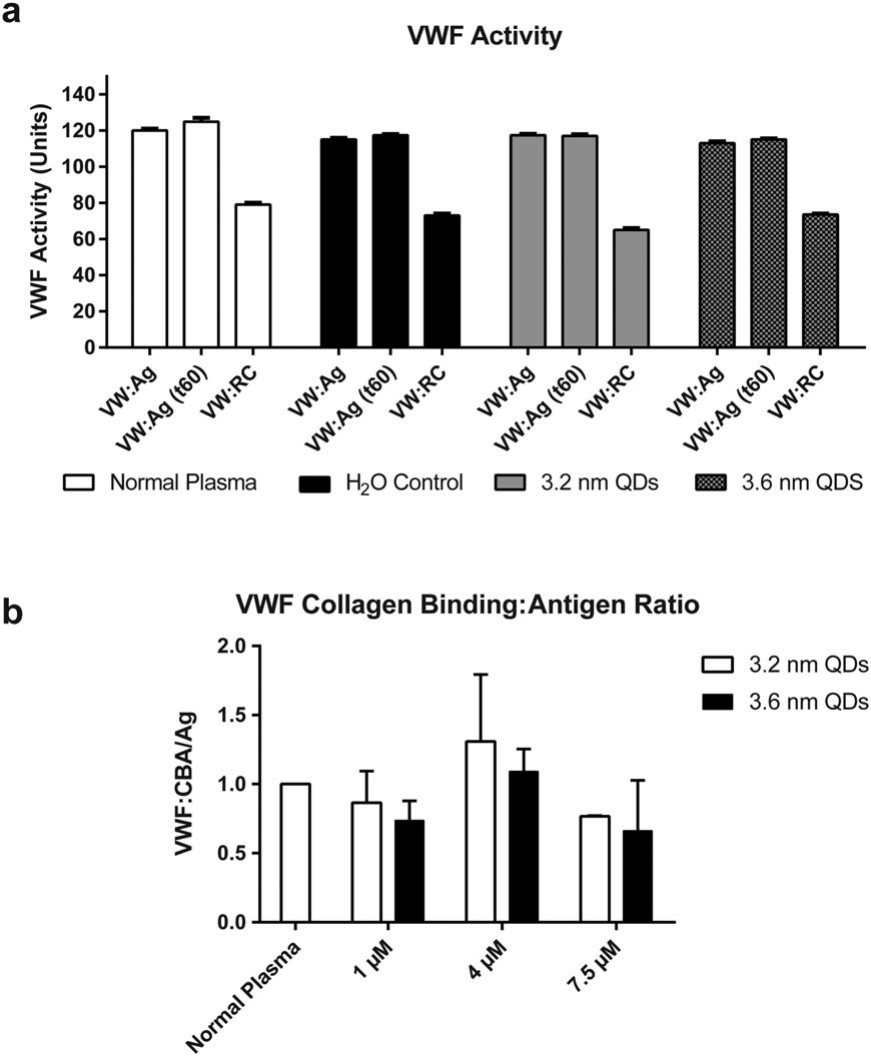
Effect of 3.2 and 3.6‐nm QDs on VWF activity. A: Von Willebrand factor activity measured following interaction with QDs at 7.5 μM, at time zero and after 1‐h incubation. The results show VWF antigen levels (VWF:Ag) and VWF ristocetin cofactor levels (VWF:RC). Data represent mean ± SEM (*N* = 3). No changes in VWF antigen or ristocetin cofactor levels are observed across all treatment groups. B: Normalised von Willebrand factor collagen binding/antigen ratios for normal plasma treated with 3.2 and 3.6 nm QDs at varying concentrations. No statistically significant changes were observed. Data represents mean ± SEM (*N* = 5).

With prolonged PFA‐100 and minimal platelet aggregation, we hypothesised that the QDs may be interacting with VWF. To investigate this, VWF antigen (VWF:Ag) level and activity were assessed in the presence and absence of QDs. In contrast to the impact on platelet aggregation, no alteration in VWF functional assays was noted with either the 3.2 or 3.6 nm QDs (Figure 3), suggesting that the abnormal PFA‐100 results and detected primary haemostatic changes are rather platelet dependent, than VWF dependent. As seen in Figure 3A, VWF:Ag remained unchanged following incubation with both 3.2 and 3.6 nm QDs. Similarly, qualitative assessment of VWF function using the VWF ristocetin co‐factor activity (VWF:RCo) and VWF collagen binding (VWF:CB) assays were not significantly altered by the incubation with QDs (Figure 3B). Previously, it has been suggested that cadmium may increase platelets responsiveness to agonists (Bhattacharya, 2002). Quantitative assessment of the function of VWF was also carried out to determine the activity of VWF following incubation with the QDs and a number of agonist. Results demonstrated that the activity of VWF was unhindered in the presence of QDs. Results of the VWF:RCo, an assay of VWF‐platelet interaction induced by the presence of the antibiotic ristocetin, again suggested normal VWF function.

### Effect of quantum dots on secondary haemostasis

#### Initial coagulation screening

**Figure 4:**
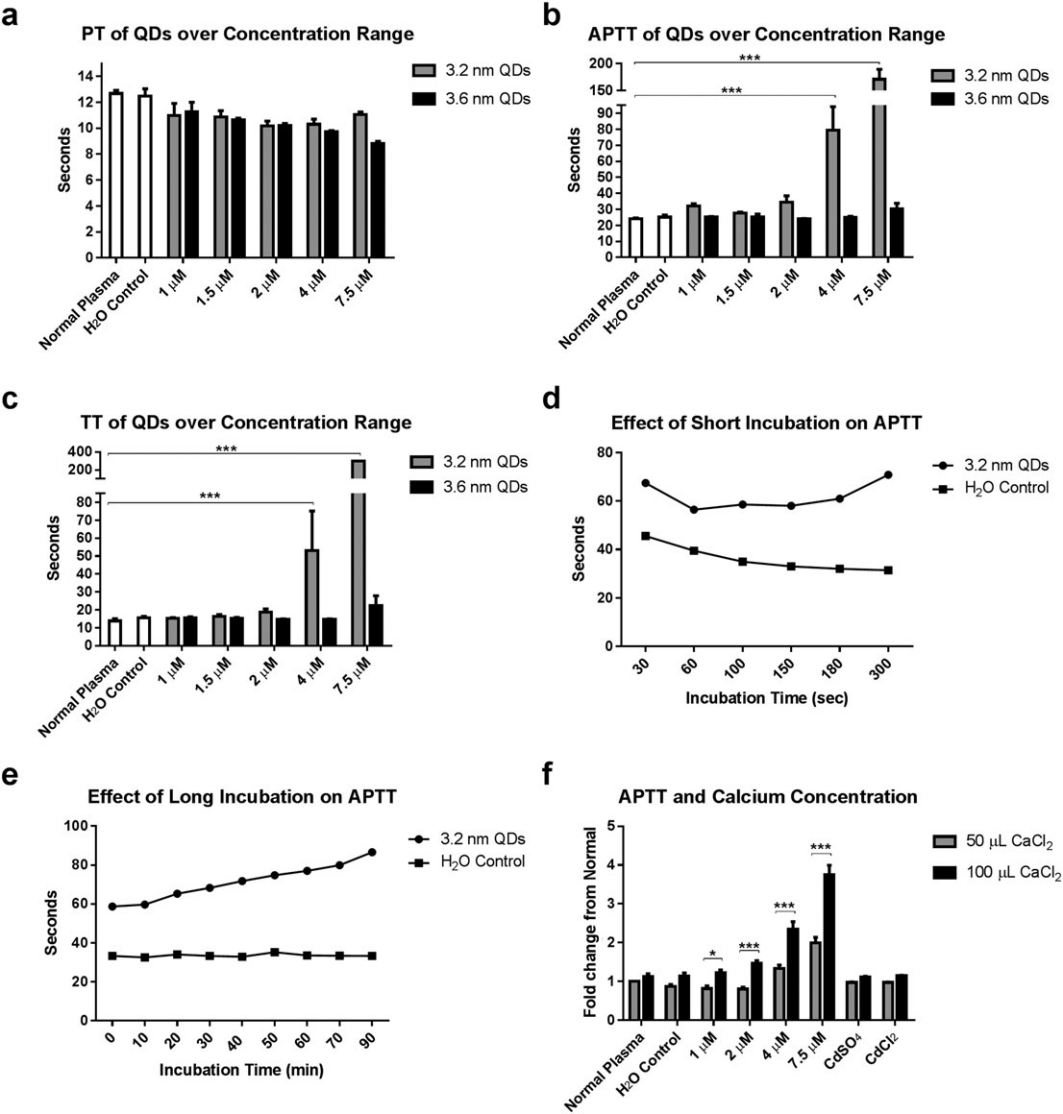
Size and concentration‐dependent effect of QDs on prothrombin time (PT), thrombin time (TT) and activated partial thromboplastin time (APTT) assays (A‐C). No change from normal was observed for the PT. Results of the APTT and TT demonstrate a profound size‐dependent intrinsic pathway centred anticoagulant effect (*** *P* < 0.001, two‐way ANOVA with Dunnett's, *n* = 4). The effect of incubation time on the APTT was also examined (D‐E). Short incubation time (D) results in APTT being sensitive to levels and activity of contact factors, with long incubation times (E) remove this sensitivity. Increasing calcium concentration also results in a further prolongation in the APTT for 3.2 nm QDs (F) (* *P* < 0.05, *** *P* < 0.001, two‐way ANOVA with Bonferroni's multiple comparison test, *n* = 4). Data represents mean ± SEM for all graphs.

In order to investigate the effect of QDs on the coagulation cascade, 3.2 and 3.6 nm QDs up to a concentration of 7.5 μM were screened using PT, TT and APTT assays, as shown in Figure 4A‐C. A size‐dependent and concentration‐dependent prolongation of the APTT was demonstrated, with the 3.2 nm QDs having the most pronounced effect. Compared to the normal control, a sevenfold (146.9 sec increase compared to untreated normal plasma, *P* < 0.001, two‐way ANOVA with Dunnett's) was observed at 7.5 μM of the 3.2‐nm QDs. A non‐significant 1.25‐fold (6.2 sec increase vs. normal control plasma, *P* = 0.78, two‐way ANOVA with Dunnett's) increase in the APTT was observed for the 3.6‐nm QDs at the same concentration. For the TT, a size‐dependent and concentration‐dependent, statistically significant prolongation was observed for the 3.2 nm QDs at concentrations in excess of 4 μM (*P* < 0.001, two‐way ANOVA with Dunnett's). No statistically significant prolongation was observed, compared to untreated normal plasma, for the 3.6 nm QDs at a concentration of 7.5 μM (*P* = 0.36, two‐way ANOVA with Dunnett's). The PT, a measure of the extrinsic factor pathway, was unchanged for either type of QD. With profound increases in clotting time for the APTT and TT assays, and no effect on the PT, these results suggest that the QDs are acting on the intrinsic pathway.

#### Impact of QDs on contact pathway

The incubation time of the APTT can alter sensitivity to different clotting factors with shorter incubation times (<2 min) reflecting contact pathway activity (Practical Haemostasis, 2014) and longer incubation times more sensitive to the distal part of the pathway (Factors VIII, IX, XI and XII). We repeated the APTT in the 3.2 nm CdTe QDs with varying incubation times, as demonstrated in Figure 4D and E, and found that APTT remained significantly prolonged over the entire incubation range, compared to the untreated plasma (*P* < 0.001, two‐way ANOVA with Sidak's multiple comparison test). These data suggest that the 3.2 nm CdTe QDs are not specifically impacting the contact pathway factors and instead exert their influence over the entire intrinsic factor pathway.

#### Intrinsic factor activity

**Figure 5:**
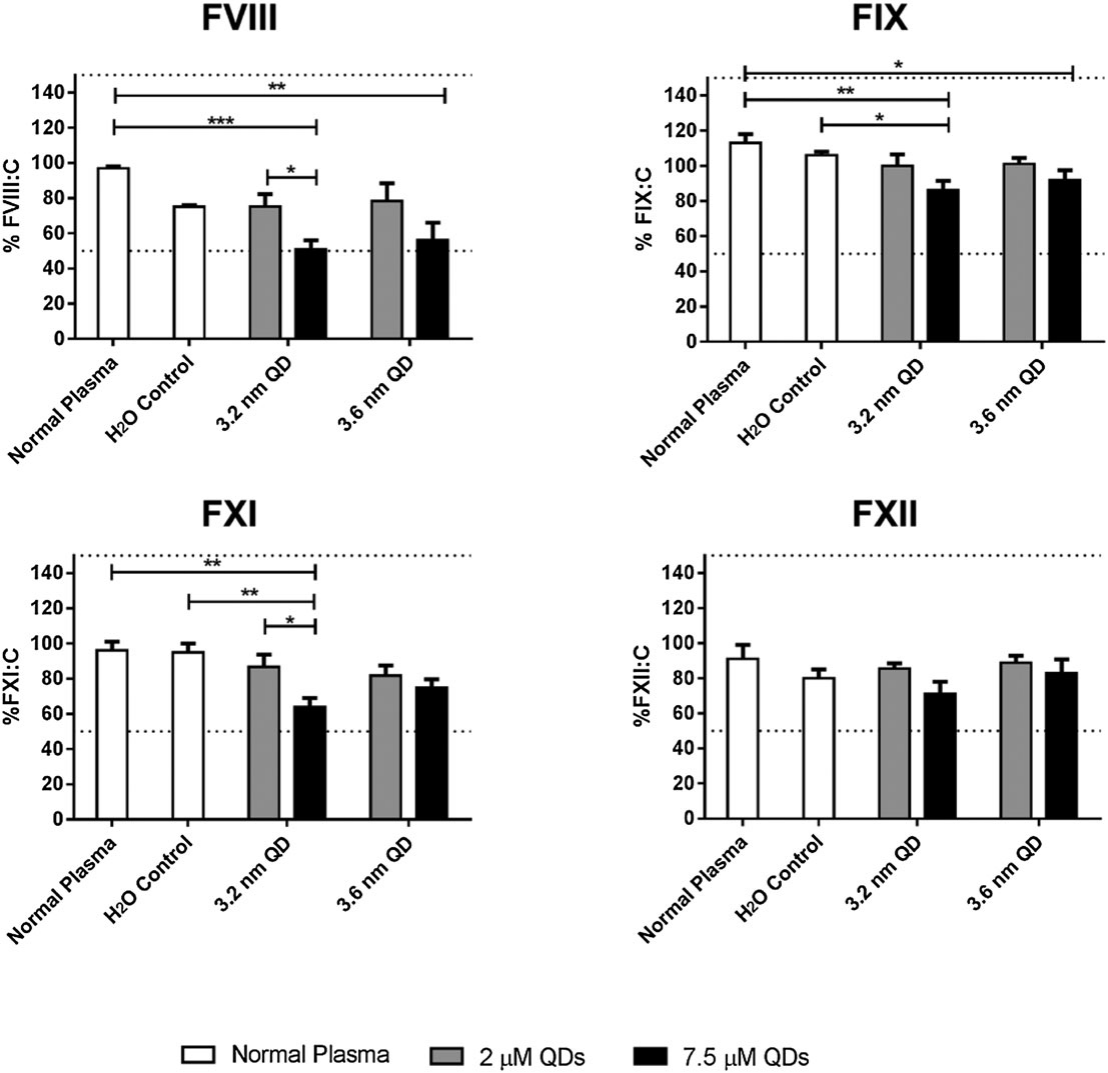
Intrinsic factor screen for activity of Factor VIII, IX, XI and XII. The IFS was carried out at 2 and 7.5 μM QD concentration. All factor activities remain within the normal clinical range of 50–150%, or 0.5–1.5 IU/mL (dashed lines), with the greatest reduction in activity being observed at 7.5 μM of the 3.2 nm QDs. Data represent mean ± SEM. * *P* < 0.05, ** *P* < 0.01, *** *P* < 0.001 ANOVA with Tukey's post‐test (*N* = 3).

Following on from the prolonged APTT results, intrinsic factor screening was carried out to identify any changes in the activity of coagulation Factors VII, IX, XI and XII (Figure 5). It should be noted that normal factor activities are typically between 50% and 150% or 0.5–1.5 IU/mL (Kadir et al., 1998; Nicoll et al., 2003). In the case of Factor VIII (FVIII:C), normal haemostasis can occur with 25% activity, and an activity of <40% of normal is required for the APTT to be prolonged (Practical Haemostasis, 2014). As shown in Figure 5, FVIII:C was reduced to 51% following incubation of plasma with 7.5 μM of the 3.2 nm QDs for 3 min. Although this FVIII:C reduction was dramatic and statistically significant (*P* < 0.001, two‐way ANOVA with Dunnett's), the observed results remained within the normal clinical range. This effect may be additive to a reduction in function in other coagulation factors or altered coagulation complex assembly. For example, Factor XI activity was reduced to 61% for the 3.2 nm QDs at 7.5 μM. Results for all other intrinsic factors screened were within their normal ranges. All the intrinsic factors were within the normal range at a concentration up to 2 μM.

#### Role of calcium

For all APTT‐based assays, PPP was incubated at intervals up to 90 min prior to initiation of coagulation with calcium. Calcium has been shown to play a crucial role in cadmium toxicity (Koçak & Akçıl, 2006; Nath et al., 1984; Washko & Cousins, 1977). Similarly, cadmium participates in a number of calcium ion‐dependent pathways due to its action as a calcium‐mimetic (Choong et al., 2014), can act to deplete circulating calcium ions (Liu et al., 2012), can replace calcium bound to cellular structures (Przelecka et al., 1991) and alter platelet aggregation (Lopez et al., 1992; Nontarach et al., 2015). To identify if leached cadmium ions from the QDs play a role in the anticoagulant effect demonstrated here, an APTT assay was carried out with an increased concentration of calcium. As shown in Figure 4F, when 100 μL of CaCl_2_ is utilised, the APTT is prolonged even further for the 3.2 nm QDs, compared to the standard assay conditions. This statistically significant prolongation is observed for all concentrations of QDs tested (*n* = 4, two‐way ANOVA with Bonferroni's comparison test). In comparison, control experiments with cadmium sulphate and cadmium chloride resulted in normal APTTs. These data suggest that it is likely a combination of the QDs' physical‐chemical properties, including their size, surface charges and potential ionic interactions, rather than cadmium atoms alone that plays the critical role. For example, the surface charge density of the 3.2 nm QDs may favour more interactions with components of the coagulation cascade, compared to that of the 3.6 nm QDs. No significant differences in the APTTs were observed with increased calcium concentration, compared to standard conditions, for normal plasma (*P* > 0.99), a volume of water equivalent to the greatest volume of QD added (7.39 μL, *P* = 0.43), cadmium sulphate (7.5 μM, *P* > 0.99) and cadmium chloride (7.5 μM, *P* > 0.99). These results demonstrate that the increased volume of calcium chloride does not dilute the plasma to a level where coagulation is unable to take place within the normal time range.

#### Impact of quantum dots on phospholipid activity

**Figure 6:**
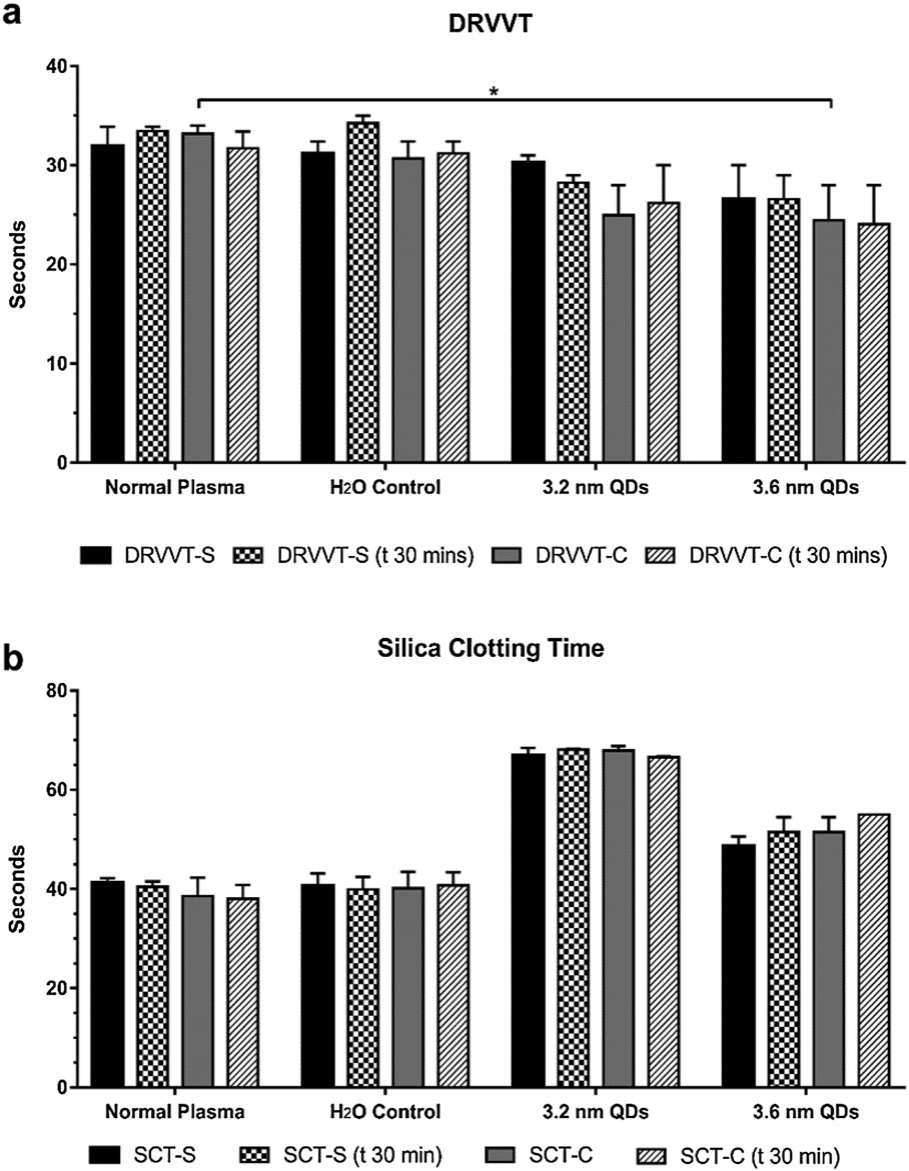
Interaction of QDs with phospholipids. A: Dilute Russell's viper venom time (DRVVT) for plasma exposed to QDs and measured at time zero and following 30 min incubation. Results are shown for high (DRVVT‐C) and low (DRVVT‐S) phospholipid concentrations). B: Silica clotting times (SCT) for plasma treated with QDs at 7.5 μM. The assay was carried out at time zero and following 30‐min incubation with high (SCT‐C) and low (SCT‐S) phospholipid concentration. Data represent mean ± SEM (*N* = 3), * *P* < 0.05, two‐way ANOVA with Dunnett's.

Similar to the ability of cadmium to alter calcium signalling and binding, it also has the ability to act as a facilitator for tissue factor binding to phospholipids (Carson & Konigsberg, 1980), and cause the exposure of phosphatidylserine (Sopjani et al., 2008). Phospholipids are essential for the formation of coagulation complexes as these assemble on the phospholipid layer of activated platelets. To this end, a number of screening tests for phospholipid function were carried out and illustrated in Figure 6. The effects of high or low phospholipid concentrations were assessed by using two phospholipid‐dependent assays, the DRVVT and SCT (Figure 6).

The DRVVT assay uses viper venom to activate Factor X with the assay being dependent on phospholipid concentrations. The assay can thus be used to identify phospholipid inhibitors. Inhibitory effects of QDs on phospholipid should impair the formation of coagulation complexes and prolong the DRVVT, with correction by saturation with excess phospholipid. As shown in Figure 6A, high and low phospholipid concentrations were used to identify if the QDs were causing a prolongation in the APTT. Shortening of the DRVVT at high phospholipid concentrations was observed for both 3.2 and 3.6 nm QDs.

No significant changes in DRVVT were observed for the 3.2 nm QDs at 7.5 μM using both, low phospholipid concentrations (DRVVT‐S) (*P* = 0.92, *n* = 3, two‐way ANOVA with Dunnett's), and high phospholipid concentrations (DRVVT‐C) (*P* = 0.06, *n* = 3, two‐way ANOVA with Dunnett's). In the case of the 3.6 nm variants at 7.5‐μM, non‐significant changes in the DRVVT‐S were observed (*P* = 0.27, *n* = 3, two‐way ANOVA with Dunnett's). A significant decrease in the DRVVT‐C was observed, however (*P* = 0.04, *n* = 3, two‐way ANOVA with Dunnett's). This reduction returned to non‐significant values following a 30‐min incubation (*P* = 0.08, *n* = 3, two‐way ANOVA with Dunnett's). As there was no recorded prolongation in the DRVVT, and the results were not dependent on incubation time, the results suggest that the alteration in APTT is not due to phospholipid interactions.

Another assay frequently used in the diagnosis of phospholipid inhibitors is the SCT. This assay is essentially an APTT assay where kaolin is replaced by silica, with the option of including low or high phospholipid concentrations. The SCT not only serves as a phospholipid‐dependent assay but also is an alternative to the APTT, utilising silica rather than kaolin as the contact activator. We therefore used the SCT as a second assay of phospholipid interaction but also to determine if the prolongation seen with the APTT was due to a kaolin‐induced artefact. As shown in Figure 6B, the SCT was also prolonged for the 3.2 nm QDs at 7.5 μM. This prolongation was independent of phospholipid concentration and incubation time. These data combined with the DRVVT and APTT results suggest that the anticoagulant effect of the QDs is not due to inhibition of coagulation complex assembly, or to any artefact brought about due to the material used to initiate coagulation.

## Conclusion

According to the results of this study, a clear anticoagulant effect is observed when plasma is treated with 3.2 nm QDs. Preliminary screen using the APTT and TT assays illustrates that the effect is centred primarily around the intrinsic pathway, with QDs yielding prolonged clotting times for these tests. The PT was unaffected across a concentration range up to 7.5 μM. Intrinsic factor screens were then carried out, and it was determined that the activity of Factors VIII, IX, XI and XII were all within the normal clinical range. The activity of FVIII was reduced to 51%, but it has been reported that an activity level of 25% will still result in normal haemostasis. As such, it is hypothesised that the anticoagulant phenomenon demonstrated in these results may be additive to less significant reductions in the activities of other coagulation factors. As stated previously, cadmium also has the ability to interfere with calcium signalling and phospholipid membrane binding, we hypothesised that prolongation of the APTT and TT may be due to the inability of the tenase or prothrombinase complexes to assemble correctly on the phospholipids required for normal coagulation. The results of the clinical assays for phospholipid function revealed that the anticoagulant properties of the 3.2 nm QDs were not due to this mechanism. Experiments with alterations to the concentrations of calcium used also yielded interesting and unexpected results. One could suggest that increasing the amount of calcium in the APTT would result in normalising the effect of the QDs and return the clotting time to the normal range. Interestingly, this was not the case as increased calcium concentrations resulted in a further increase in the APTT. At a concentration of 7.5 μM of the 3.2 nm QDs, the APTT is shown to be twice that of normal at the standard 50 μL calcium concentration. This is increased to 3.75 times from normal when an increased volume of calcium is used. No changes in the clotting times were observed under untreated control conditions, plasma treated with an equivalent volume of water as in QDs samples or plasma treated with a 7.5 μM solution of cadmium salts, indicating that this effect is strictly due to the presence of QDs and not cadmium ions as originally hypothesised.

**Figure 7:**
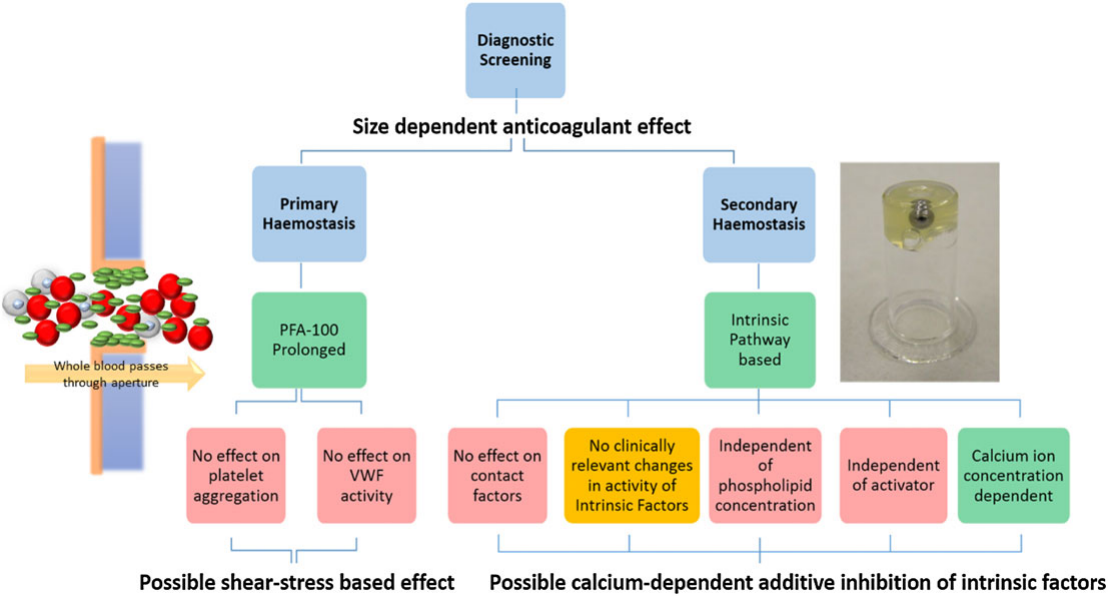
3.2‐nm cadmium telluride quantum dots (CdTe QDs) interact with a number of components involved in haemostasis. In a shear‐based platelet function analyser system, 3.2 nm QDs cause the prolongation in platelet aggregation time. Under static conditions, it was determined that the QDs do not inhibit platelet aggregation or VWF activity. The effect of the QDs was centred on the intrinsic pathway of the coagulation cascade. The anticoagulant properties observed are calcium ion concentration dependent, and activator‐type and phospholipid concentration independent. No effect was observed that may be due to interaction with the contact factors, and the activities of the intrinsic factors were within normal clinical ranges. The observed anticoagulant activities of the QDs may be due to an additive effect of somewhat reduced Factor VIII activity and inhibited coagulation complex formation. Colour coding: Green – confirmed effect; Red – no effect; Yellow – possible effect.

In conclusion, the results of the clinically relevant diagnostic assays indicate that the 3.2 nm QDs affect coagulation through a not yet fully understood mechanism, which based on our study does not align fully with the mechanisms of toxicity associated with free elemental or ionic cadmium. What is evident, however, is that the anticoagulant phenomenon is centred on the intrinsic coagulation pathway and that calcium ion concentration plays a crucial role, as summarised in Figure 7. It may be possible that the results observed are due to an additive effect of the QDs through the lowered Factor VIII activity combined with reduction in other factors' activity, altered thrombin generation and possible VWF or phospholipid interactions. Similarly, the 3.2 nm QDs may also be able to bind to or interact with key elements of the coagulation factors or phospholipids altering their function, whereas the 3.6 nm QDs stand above this critical size rendering them unable to bind these structurally or functionally crucial components. Further molecular modelling and binding studies may provide more evidence for this.

## Conflict of Interest and Disclosures

M.L. has received research funding from Baxalta and has served on advisory boards for Baxalta. J.S.O'D has served on the speaker's bureau for Baxter, Bayer, Novo Nordisk, Boehringer Ingelheim, Leo Pharma and Octapharma. He has also served on the advisory boards of Baxter, Bayer, Octapharma CSL Behring, Daiichi Sankyo, Boehringer Ingelheim and Pfizer. J.S.O.D has also received research grant funding awards from Baxter, Bayer, Pfizer and Novo Nordisk.
